# 80 Donor Skin Reduction in Full-thickness Wounds Using Autologous Cell Harvesting Device with Meshed Autograft

**DOI:** 10.1093/jbcr/irae036.079

**Published:** 2024-04-17

**Authors:** Jeffrey W Shupp, Lisa Rae, Derek Bell, Theresa L Chin, Alfredo C Cordova, Kevin N Foster, Lourdes Castanon

**Affiliations:** MedStar Health |Georgetown University School of Medicine, Washington, DC, DC; Temple University Hospital, Philadelphia, Pennsylvania; University of Rochester, Rochester, New York; Department of Surgery, Division of Trauma, Burns and Surgical Critical Care, University of California, Irvine School of Medicine, Orange, CA; Sarasota Memorial Hospital, Sarasota, Florida; Arizona Burn Center Valleywise Health, Phoenix, AZ; University of Arizona, Tucson, Arizona; MedStar Health |Georgetown University School of Medicine, Washington, DC, DC; Temple University Hospital, Philadelphia, Pennsylvania; University of Rochester, Rochester, New York; Department of Surgery, Division of Trauma, Burns and Surgical Critical Care, University of California, Irvine School of Medicine, Orange, CA; Sarasota Memorial Hospital, Sarasota, Florida; Arizona Burn Center Valleywise Health, Phoenix, AZ; University of Arizona, Tucson, Arizona; MedStar Health |Georgetown University School of Medicine, Washington, DC, DC; Temple University Hospital, Philadelphia, Pennsylvania; University of Rochester, Rochester, New York; Department of Surgery, Division of Trauma, Burns and Surgical Critical Care, University of California, Irvine School of Medicine, Orange, CA; Sarasota Memorial Hospital, Sarasota, Florida; Arizona Burn Center Valleywise Health, Phoenix, AZ; University of Arizona, Tucson, Arizona; MedStar Health |Georgetown University School of Medicine, Washington, DC, DC; Temple University Hospital, Philadelphia, Pennsylvania; University of Rochester, Rochester, New York; Department of Surgery, Division of Trauma, Burns and Surgical Critical Care, University of California, Irvine School of Medicine, Orange, CA; Sarasota Memorial Hospital, Sarasota, Florida; Arizona Burn Center Valleywise Health, Phoenix, AZ; University of Arizona, Tucson, Arizona; MedStar Health |Georgetown University School of Medicine, Washington, DC, DC; Temple University Hospital, Philadelphia, Pennsylvania; University of Rochester, Rochester, New York; Department of Surgery, Division of Trauma, Burns and Surgical Critical Care, University of California, Irvine School of Medicine, Orange, CA; Sarasota Memorial Hospital, Sarasota, Florida; Arizona Burn Center Valleywise Health, Phoenix, AZ; University of Arizona, Tucson, Arizona; MedStar Health |Georgetown University School of Medicine, Washington, DC, DC; Temple University Hospital, Philadelphia, Pennsylvania; University of Rochester, Rochester, New York; Department of Surgery, Division of Trauma, Burns and Surgical Critical Care, University of California, Irvine School of Medicine, Orange, CA; Sarasota Memorial Hospital, Sarasota, Florida; Arizona Burn Center Valleywise Health, Phoenix, AZ; University of Arizona, Tucson, Arizona; MedStar Health |Georgetown University School of Medicine, Washington, DC, DC; Temple University Hospital, Philadelphia, Pennsylvania; University of Rochester, Rochester, New York; Department of Surgery, Division of Trauma, Burns and Surgical Critical Care, University of California, Irvine School of Medicine, Orange, CA; Sarasota Memorial Hospital, Sarasota, Florida; Arizona Burn Center Valleywise Health, Phoenix, AZ; University of Arizona, Tucson, Arizona

## Abstract

**Introduction:**

The Autologous Cell Harvesting Device is a medical device designed for regenerative medicine applications. Its primary function is to enable clinicians to prepare an autologous skin cell suspension (ASCS) from a small sample of a patient's skin at the point of care. This innovative approach allows for the use of a patient's own skin cells in various medical procedures. The ASCS device received Food and Drug Administration (FDA) approval in September 2018 for the treatment of acute thermal burn wounds, demonstrating ASCS as an effective tool for promoting tissue regeneration and wound healing while significantly reducing donor skin requirements. This study was designed to evaluate safety and effectiveness of ASCS in conjunction with widely meshed autografts in patients undergoing reconstruction of full-thickness, non-thermal skin defects such as those resulting from trauma and surgery.

**Methods:**

This prospective, randomized, within-subject study enrolled patients ≥5 years of age with acute non-thermal full-thickness skin wounds requiring autografting. Investigators were responsible for their respective autografting plans for closure in alignment with their stand of care. Comparable treatment areas of >80 cm2 each were randomized to receive autografting treatment per investigator’s pre-identified plan (Control), or ASCS treatment in combination with a more widely meshed autograft than that of the Control. Co-primary effectiveness endpoints included 100% healing at (or prior to) 8 weeks post-treatment confirmed at 2 consecutive study visits at least 2 weeks apart, and the ratio of donor site to treatment area expansion ratios.

**Results:**

Of the 65 patients enrolled, 63% (n = 41) had surgical skin defects while 37% (n = 24) had traumatic skin defects. Week 8 healing of the ASCS-treated areas (65%) was non-inferior to the Control areas (58%) (p < 0.025) and donor skin sparing in the ASCS-treated was superior to the Control area, using 27% less donor skin, on average (p < 0.025), meeting both co-primary endpoints.

**Conclusions:**

ASCS application combined with a widely meshed autograft for the treatment of non-thermal full-thickness wounds required significantly less donor skin for closure and had comparable healing outcomes when compared to standard of care. The Autologous Cell Harvesting Device demonstrated its effectiveness in soft tissue wound repair by reducing the amount of donor skin and maintaining consistent healing outcomes.

**Applicability of Research to Practice:**

The Autologous Cell Harvesting Device offers a clinically advantageous approach to autografting by reducing the amount of skin needed for closure without compromise to healing outcomes.

